# Complete Genome Sequence of Indigo-Producing Bacterium *Celeribacter* sp. Strain TSPH2

**DOI:** 10.1128/genomeA.01124-17

**Published:** 2017-11-09

**Authors:** Hae-Seon Kim, Sun Ho Cha, Ho Young Suk, Tae-Hyung Kwon, Jung-Hee Woo

**Affiliations:** a Gyeongbuk Institute for Marine Bio-Industry (GIMB), Uljin, Gyeongbuk, South Korea; b GenoTech Corporation, Daejeon, South Korea; c Department of Life Sciences, Yeungnam University, Gyeongsan, Gyeongbuk, South Korea; d Chuncheon Bioindustry Foundation, Chuncheon, South Korea

## Abstract

*Celeribacter* sp. strain TSPH2, a novel producer of indigo, was isolated from oil-contaminated sediment. We present here its genome sequence consisting of one circular chromosome (4 Mb) and one plasmid (0.15 Mb), with an overall G+C content of 60.9%. This strain contains oxygenase genes involved in indigo synthesis, such as flavin-containing monooxygenase.

## GENOME ANNOUNCEMENT

Indigo, one of the oldest blue textile dyes, has traditionally been produced from the extracts of several plants, such as *Indigofera* sp. and *Polygonum tinctorium*. In recent decades, indigo has been generally produced by chemical synthesis, which poses risks to the health of humans and the environment. As a result of attempts to develop microbiological methods for the production of environmentally friendly indigo, several wild-type strains producing indigo, such as strains belonging to the genera *Pseudomonas*, *Acinetobacter*, and *Comamonas*, have been found to date (1–5). Indigo can also be produced by recombinant *Escherichia coli* expressing various oxygenases (6–9). We recently isolated the indigo-producing strain *Celeribacter* sp. TSPH2 and report here its genome information.

Strain TSPH2 was isolated from a sample of oil-contaminated sediment collected on 11 May 2011 in the city of Taean, which is located on the west coast of South Korea. This strain was identified by 16S rRNA gene sequence analysis with 99% confidence. The extracted DNA was used to construct 20-kb SMRTbell template libraries. The whole-genome sequence was determined using the PacBio RS II sequencing platform (Pacific Biosciences) (10), yielding 151,110 long reads totaling 774,000,765 bp after filtering of the subreads. *De novo* assembly was conducted using the Hierarchical Genome Assembly Process version 2.3 (11), including consensus polishing with Quiver. As the estimated genome size was 4,212,407 bp with an average coverage of 123×, we performed error correction on the longest seed bases (about 30×, 150,019,215 bp) and then assembled the rest of the shorter reads with the error-corrected reads. Since bacterial genomes and plasmids are typically circular, each of the contigs was checked using MUMmer version 3.5 (12). The finished genomic sequences were annotated with NCBI’s Prokaryotic Genome Annotation Pipeline.

The *Celeribacter* sp. TSPH2 genome contained one circular chromosome of 4,009,276 bp with a G+C content of 61.3% and one circular plasmid of 148,161 bp with a G+C content of 60.4%. The genome contained 9 rRNAs, 50 tRNAs, and 4,215 protein-coding genes.

Our genome analysis revealed that this strain carried oxygenases, such as flavin-containing monooxygenase (FMO), involved in the synthesis of indigo using indole as the substrate. The FMO exhibited a similarity of 80% to that observed in *Methylophaga aminisulfidivorans* (13).

### Accession number(s).

The complete genome sequence of *Celeribacter* sp. strain TSPH2 has been deposited in GenBank under the accession numbers CP022196 and CP022197. *Celeribacter* sp. TSPH2 is currently available from the Korean Culture Center of Microorganisms under the accession number KCCM 11874P.
